# Manual of Pathology, Including Bacteriology, the Technique of Post Mortems and Methods of Pathologic Research

**Published:** 1898-05

**Authors:** 


Manual of Pathology, including Bacteriology, the Technique of Post Mortems and Methods of Pathologic Research. By W. M. Late Coplin, M. D., Professor of Pathology and Bacteriology, Jefferson Medical College and Pathologist to Jefferson Medical College Hospital and to the Philadelphia (Blockley) Hospital; Bacteriologist to the Pennsylvania State Board of Health. Second edition. Small octavo, pp. xxi.—638. With 268 illustrations. Philadelphia: P. Blakiston, Son & Co., 1012 Walnut street. 1897.

This volume is the outgrowth of a series of abstracts of the author's lectures, delivered at the Jefferson medical college during the winter of 1894 and 1895. The original volume was very soon exhausted and encouraged greatly thereby, Coplin decided to revise, enlarge and complete the work and the present volume is the result. The author divides the work into three parts, Part I. being devoted to technic, Part II. to general pathology and Part III. to special pathology.

Under Part I. the author discusses the method of making post mortem examinations, the various histologic methods, microscopic examination of the urine, technic of sputum examinations, technic of blood examinations. These chapters are all well written, full, yet concise and embrace all the newer methods of microscopical research.

The chapters of Part II. deal with the degenerations and infiltrations, necroses, circulatory disturbances, inflammations, tumors, infective granulomata and a very good exposition of general bacteriology. The animal parasites, different intoxications and temperature changes are also discussed under this head.

Part III. takes up the pathology of the various organs and systems of the body, beginning with the blood.

The illustrations, 268 in number, are, as a whole, not what one might expect. Some, indeed, are excellent, but some are so diagrammatic and simplified that they convey little impression as to the real condition. This applies to some of the tumors.

On the whole, the author has succeeded in bringing forth a very laudable and instructive work and one that will find a place in many laboratories The presswork, paper and binding are good examples of the printer's art. W. C. K.

